# The child brain computes and utilizes internalized maternal choices

**DOI:** 10.1038/ncomms11700

**Published:** 2016-05-24

**Authors:** Seung-Lark Lim, J. Bradley C. Cherry, Ann M. Davis, S. N. Balakrishnan, Oh-Ryeong Ha, Jared M. Bruce, Amanda S. Bruce

**Affiliations:** 1 Department of Psychology, University of Missouri-Kansas City, 5030 Cherry Street, Kansas City, Missouri 64110, USA; 2 Department of Pediatrics, University of Kansas Medical Center, 3901 Rainbow Boulevard, Kansas City, Kanas 66160, USA; 3 Center for Children's Healthy Lifestyles & Nutrition, 610 East 2nd Street, Kansas City, Missouri 66108, USA; 4 Department of Mechanical and Aerospace Engineering, Missouri University of Science and Technology, 194 Toomey Hall, Rolla, Missouri 65409, USA; 5 School of Engineering and Computer Science, Shiv Nadar University, NH91, Tehsil Dadri, Gautam Buddha Nagar, Uttar Pradesh 201314

## Abstract

As children grow, they gradually learn how to make decisions independently. However, decisions like choosing healthy but less-tasty foods can be challenging for children whose self-regulation and executive cognitive functions are still maturing. We propose a computational decision-making process in which children estimate their mother's choices for them as well as their individual food preferences. By employing functional magnetic resonance imaging during real food choices, we find that the ventromedial prefrontal cortex (vmPFC) encodes children's own preferences and the left dorsolateral prefrontal cortex (dlPFC) encodes the projected mom's choices for them at the time of children's choice. Also, the left dlPFC region shows an inhibitory functional connectivity with the vmPFC at the time of children's own choice. Our study suggests that in part, children utilize their perceived caregiver's choices when making choices for themselves, which may serve as an external regulator of decision-making, leading to optimal healthy decisions.

## Abstract (web summary)

Mothers advocate eating healthy foods while children like to eat tasty foods. Lim and colleagues demonstrate that children incorporate their mothers' food choices while deciding what to eat as well as provide the neural correlates of this decision making process.

## Graphical abstract

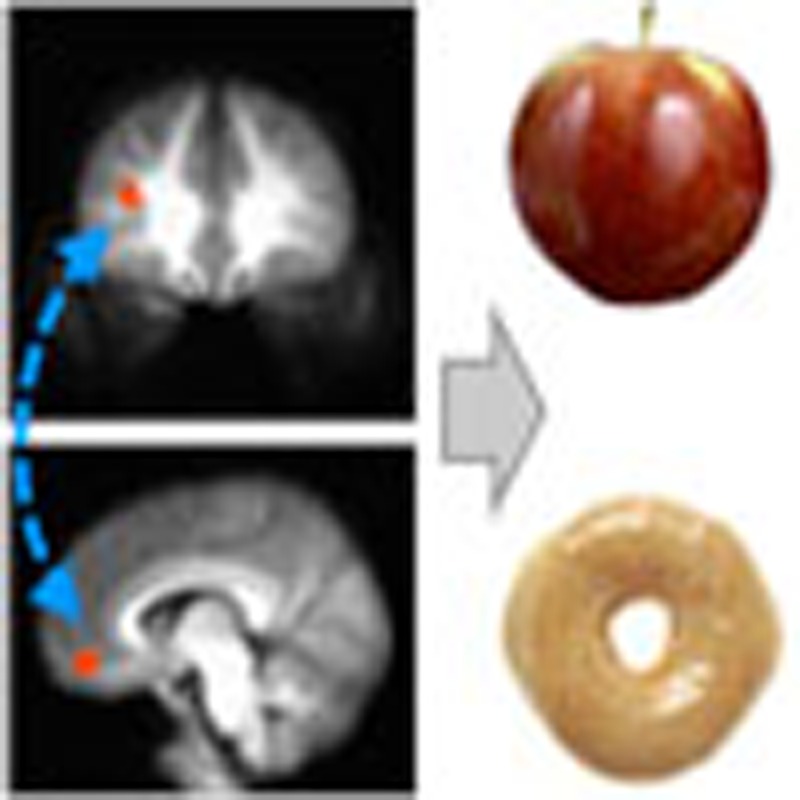

Through early development, animals and humans learn how to make optimal choices (for example, what to eat and when to sleep). Caregivers initially control the most important decisions, but children gradually begin to make independent choices. Making effective decisions is critical for survival and well-being. Child food decisions are of particular importance as they establish lifelong health behaviour patterns. Given the prevalence of childhood obesity^1^, it is critical to better understand how children make food choices. Encouraging healthy food decisions (for example, choosing vegetables over dessert at the school cafeteria) is a daunting endeavour. Owing to an innate proclivity for sweet and salty tastes, children have strong preferences for foods that are extensively processed and calorically dense. Furthermore, children have poor or limited explicit knowledge of nutrition information about delicious but unhealthy foods^2^.

To make healthy food decisions, people must assign priority to healthiness that provides long-term nutritional benefits, rather than good taste that produces short-term pleasure. In other words, voluntary healthy choices require effortful and goal-directed self-regulation that involves cognitive control, inhibition, set-shifting, planning and future thinking^3^. These abilities are not fully matured in childhood^4^. The protracted structural and functional maturation of the prefrontal cortex that is responsible for these executive functions can restrict a child's ability to make future goal-oriented decisions^5^. Although adults are better able to consider both tastiness and healthiness when making food choices^6^,^7^,^8^, children may not make food decisions that actively incorporate both tastiness and healthiness due to maturational cognitive limitations. Despite these developmental constraints, children still learn important skills and values by observing and modelling parents or caregivers' behaviours^9^. Parents can act as external regulators until children become gradually more capable of regulating their own decisions that emphasize long-term goals rather than immediate pleasure^10^. Through repeated child–parent interactions, children learn and internalize parental expectations. When children are asked to make their own choices, they may not fully incorporate abstract information such as the health value of the food. Yet, children may guess what their parents would do for them in the same situation, and utilize this information to inform their own choices. Children may use internalized maternal preferences (for example, would my mom want me to eat this food?) as a compensatory decision-making tool until they fully develop independent self-regulatory mechanisms in early adulthood. In other words, utilizing internalized maternal choices may help children to make better decisions while overcoming the cognitive limitations or bounded rationality^11^ of childhood. We can examine this decision-making process using computational models and functional neuroimaging.

Although children's decision-making may be critically different from that of adults, little is known about its computational and neurobiological mechanisms, particularly in regard to food choices. We hypothesized that children's food preferences would be primarily determined by the tastiness of a food. The delicious taste of a food (for example, sweet unhealthy treats) provides immediate pleasure, rather than a future-oriented state of good health. Nonetheless, we hypothesized that based on repetitive child–parent interactions, children would know which foods their parents prefer them to eat. Thus, we expected that children's food decisions would be guided by their own preferences (that is, what they like) as well as their projected maternal choices (that is, what children believe their mom would want them to eat). Utilizing this dual process of food decision-making (for example, choosing a healthier snack at the school cafeteria after considering what their mom would likely choose for them) can provide nutritional advantages for children who do not have fully maturated cognitive or self-regulation functions. Recent decision neuroscience studies demonstrate that the human brain infers and tracks other people's decisions in various social situations, including the presence of other people^12^,^13^,^14^.

In the current study, we tested two hypotheses by combining behavioural tests and functional magnetic resonance imaging (fMRI) data. First, we hypothesized that children's food decisions would be determined by their own preferences and their projected mom's choices for them. This makes children's decision-making qualitatively different from adults' decision-making (see the ‘projected mom's decision' term in the equation below for our model of children's food choices). Second, we hypothesized that the child's brain would encode these two decision variables (that is, own preference and projected mom's preference) at the time of children's own choices. On the basis of previous neuroimaging literature^6^,^7^,^15^,^16^,^17^, we predicted children's own preferences would be encoded in the ventromedial prefrontal context (vmPFC) and the projected mom's choices would be encoded in the left dorsolateral prefrontal cortex (dlPFC).


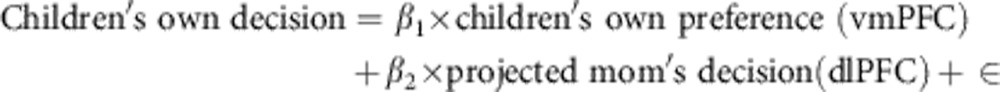


## Results

### Participants and procedures

**Figure 1:**
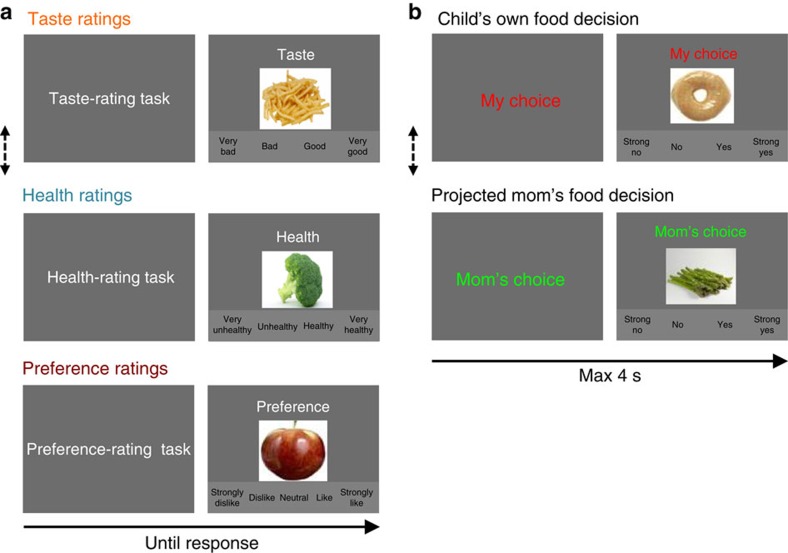
Experimental tasks. (**a**) Children completed taste (four-point scale: very bad–very good), health (four-point scale: very unhealthy–very healthy) and overall preference (five-point scale: strongly dislike–strongly like) ratings for 60 different food items before fMRI scans. All food images used are in the public domain.). All error bars denote s.e. The order of taste and health ratings was counterbalanced across subjects. (**b**) During fMRI scans, children made food decisions in own choice and mom's choice conditions (four-point scale: strong no–strong yes). In mom's choice condition, children were asked to guess their mom's food choices for them. Own choice and mom's choice blocks were randomly presented.

Twenty-five children between the ages of 8 and 14 years (mean 10.9 years old; 14 boys) and their mothers were enrolled in the study. Children were instructed to fast for 3 h before the experiment to ensure moderate and similar levels of hunger. After obtaining informed consent and assent, children provided separate taste (four-point scale; very bad–very good) and health (very unhealthy–very healthy) ratings, as well as their overall preference (five-point scale; strongly dislike–strongly like) ratings for 60 different food items that varied on taste and health attributes outside the scanner (Fig. 1a). Next, while undergoing fMRI scans, children made a series of choices for each food item shown on the screen in two different experimental conditions that were randomly presented (Fig. 1b). In the ‘own choice' condition, children were asked to make their own food decisions based on how much they wanted to eat that food. In the ‘mom's choice' condition, children were asked to estimate what their moms would choose for them to eat. In both conditions (120 choices total), children entered their decision values by using a four-button response pad (strong no, no, yes and strong yes). For a better understanding of our data, descriptive statistics and pairwise correlations of behavioural ratings are reported in Supplementary Table 1.

### Behavioural results

**Figure 2:**
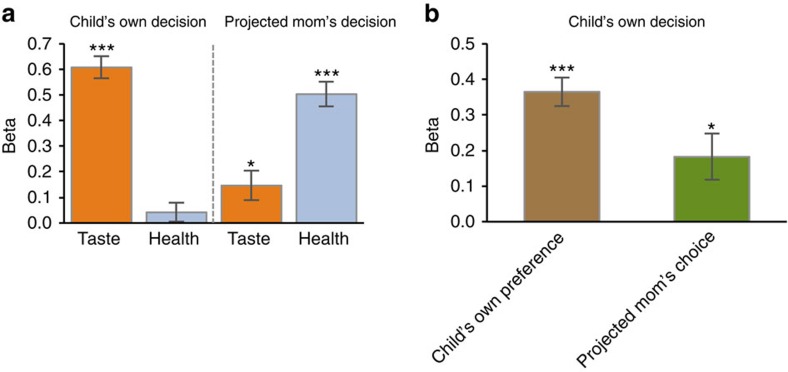
Children's food decision. (**a**) Children's own food choices were solely predicted by taste ratings, whereas the projected mom's food choices were predicted by both taste and health ratings. (**b**) Children's own food choices were predicted by both own preferences (from behavioural ratings) and the projected mom's choices (estimated from fMRI mom's choice condition). All error bars denote s.e. *P<.05; ***P<0.001.

Before the fMRI data, we first examined how children incorporate taste and health values into their own choices and their projected mom's choices as well. We predicted that children would make their own food decisions primarily using taste values rather than health values. We fitted a linear regression model of taste and health ratings on children's decisions separately for each experimental condition. In this regression model, two rating predictor variables were simultaneously entered. Then, we performed *t*-tests with estimated regression coefficients for the group-level analyses. As hypothesized, only taste ratings (mean *β*=0.61, *t*_24_=14.40, *P*<0.001), and not health ratings (mean *β*=0.04, *t*_24_=1.11, *P*=0.28), significantly predicted children's own food decisions. However, both taste ratings (mean *β*=0.15, *t*_24_=2.57, *P*<0.05) and health ratings (mean *β*=0.50, *t*_24_=10.22, *P*<0.001) significantly predicted the children's projected mom's food decisions for them (Fig. 2a). This result suggests that children do not utilize health information for their own food choices, despite possessing the knowledge of nutritional health values. However, they predict that their mothers will use health information when making food choices for them. None of the taste and health rating beta-weights showed a significant correlation with age, pubertal development or body mass index (BMI) *z*-scores in our sample (correlation coefficients were checked separately at *P*<0.05).

For the food choice data acquired during fMRI scans, we first compared the reaction times between children's own choice and mom's choice conditions. Not surprisingly, children made their own choices significantly quicker than mom's choices (own choice mean response time (RT)=1.63 s, s.d.=0.24; mom's choice mean RT=1.76 s, s.d.=0.76; *t*_24_=5.05, *P*<0.001), suggesting that controlled decision-making procedures (that is, the projected mom's choices) require additional cognitive resources. Also, a significant positive correlation between children's own and mom's choice RTs was observed across participants (*r*=0.90, *P*<0.001). Next, we checked the percentages of children's healthy decisions separately for each decision condition. Healthy decisions were defined by ‘yes' or ‘strong yes' decisions for healthy food items (based on children's subjective health ratings outside the scanner) and ‘no' or ‘strong no' decisions for unhealthy food items (based on children's subjective health ratings outside the scanner). Not surprisingly, children made relatively less healthy choices for their own decision trials (*M*=48.4%, s.d.=15.6%) compared with the projected mom's decision trials (*M*=76.9%, s.d.=13.0%; *t*_24_=9.13, *P*<0.001). Similarly, we examined the percentages of children's tasty decisions. Tasty decisions were defined by ‘yes' or ‘strong yes' decisions for tasty food items (based on children's subjective taste ratings outside the scanner) and ‘no' or ‘strong no' decisions for not-tasty food items (based on children's subjective taste ratings outside the scanner). As expected, children made relatively more tasty choices for their own decision trials (*M*=85.5%, s.d.=6.5%) compared with the projected mom's decision trials (*M*=59.9%, s.d.=15.2%; *t*_24_=8.94, *P*<0.001). Next, we checked the percentage of children's self-regulated decisions separately for each decision condition. The self-regulated decisions were operationally defined by ‘yes' or ‘strong yes' decisions for healthy but not-tasty food items (based on children's subjective taste and health ratings outside the scanner) and ‘no' or ‘strong no' decisions for tasty but unhealthy food items (based on children's subjective taste and health ratings outside the scanner). Again, we found that children made relatively less self-regulated food choices for own decision trials (*M*=17.1%, s.d.=15.6%) compared with the projected mom's decision trials (*M*=67.6%, s.d.=22.8%; *t*_24_=11.69, *P*<0.001). None of the percentages of healthy decisions, tasty decisions and self-regulated decisions was significantly correlated with age, puberty development or BMI *z*-scores. However, the children with higher self-control scores from a self-report questionnaire showed fewer self-regulated decision ratio differences between the two conditions (the projected mom's choices–own choices; *r*=−0.45, *P*<0.05), suggesting that the decision context had less impact on the children with higher self-regulation ability. Similarly, the children who showed smaller decision time differences (mom's choices–own choices) made fewer self-regulated decisions in the own choice condition (*r*=−0.45, *P*<0.05).

Most importantly, we hypothesized that children who did not incorporate health values into their own decisions would still utilize the projected moms' food choices for them when they made their own food decisions. In other words, we hypothesized that children's food decisions would be determined by both the projected mom's choices, as well as their own preferences. To test this main hypothesis of the computational model of children's food decisions, we fitted a linear regression model on the children's own food decision data (inside the scanner). For each food item choice, the children's own preferences (from overall liking ratings outside the scanner) and the children's projected mom's choices for them (from the mom's choice data inside the scanner) were simultaneously entered into the regression model to predict children's own food decisions. Interestingly, the projected mom's choices (mean *β*=0.18, *t*_24_=2.79, *P*<0.05), as well as children's own preferences (mean *β*=0.36, *t*_24_=9.12, *P*<0.001), significantly predicted children's own food decisions (Fig. 2b), suggesting that both decision variables uniquely explain children's own food decisions after controlling for each other. Across participants, both beta-weights were not correlated with age, pubertal development or BMI *z*-scores.

To further establish the robustness of our findings of the projected mom's choices in predicting children's own food decisions, we performed an additional confirmatory regression model on children's own choices that included additional health and taste ratings, as well as the child's own preferences and the projected mom's choices. To avoid a potential co-linearity issue due to high correlations between children's own preferences and taste ratings (*r*=0.70, *P*<0.01), additional taste and health rating predictors were orthogonalized in respect to both children's own preference and the projected mom's choices in this regression model. Again, the beta-weights of the projected mom's choices were statistically significant (mean *β*=0.24, *t*_24_=3.99, *P*<0.001), even after controlling for taste and health ratings (mean *β*=0.34, *t*_24_=9.13, *P*<0.001; mean *β*=−0.06, *t*_24_=−3.02, *P*<0.01), as well as their own preferences (mean *β*=0.44, *t*_24_=10.40, *P*<0.001). Interestingly, the health ratings showed a significant negative beta-weight (that is, higher healthiness predicts ‘not to eat' decisions) after controlling for the projected mom's choices in this model. This finding corresponded to a significant zero-order correlation between children's own preferences and health ratings (*r*=−0.17, *P*<0.05). Furthermore, the projected mom's choices still significantly predicted children's own food decisions even when we conducted similar regression models separately for healthy and unhealthy food items (mean *β*=0.28, *t*_24_=3.89, *P*<0.005; mean *β*=0.25, *t*_24_=3.09, *P*<0.01). The child's health ratings were not significant in the models. This indicates the projected mom's choices uniquely explained the children's food decisions that could not be simply substituted by children's healthy ratings. Overall, children's choice data strongly support our computational decision model in which both the projected mom's choices and children's own preferences determine the children's own food decisions.

### fMRI results

**Figure 3:**
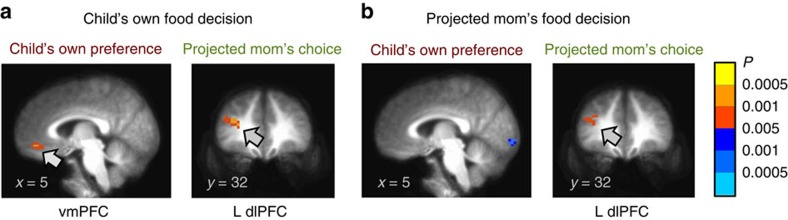
vmPFC and dlPFC activations at the time of choices. (**a**) In own choice trials, activity in vmPFC positively correlated with children's own preference ratings and activity in left dlPFC positively correlated with the projected mom's choices. (**b**) In mom's choice trials, activity in left dlPFC positively correlated with the projected mom's choices. All images were thresholded at *P*<0.05 corrected.

We examined fMRI data to identify brain regions that encode these two decision variables representing children's own preferences and their projected mom's choices, which were both found to significantly predict children's behavioural food decisions. Similar to the behavioural regression model, the parametric regressors of children's own food preferences and the projected mom's choices were simultaneously entered into the general linear model (GLM) of fMRI data along with other regressors of non-interest. Consistent with our hypotheses of the neural model of children's food decisions, when children made their own food choices, brain activity in the vmPFC positively correlated with own preferences, and brain activity in the left dlPFC positively correlated with the projected mom's choices (*P*<0.05 corrected; Fig. 3a). Even though our main goal was to confirm these two critical decision variables at the time of children's own choices with fMRI data, we further explored the neural correlates of these variables at the time of the projected mom's choices. Interestingly, when children estimated their mom's food choices for them, the same left dlPFC area showed a positive correlation with the projected mom's choices, but the vmPFC area did not show a significant correlation (*P*<0.05 corrected; Fig. 3b and Table 1). The lack of the vmPFC activity that encoded children's own preference at the time of the projected mom's choice trials could be explained by the incentive structure of our task (that is, no motivational reason to encode expected rewards, because no food item was selected from mom's trials and given to children). However, the overlapping dlPFC activity suggests that children might use identical neural mechanisms to compute the projected mom's choices in both decision conditions. In addition, we inspected two event indicator regressors to explore potential systematic task differences (for example, motivational or cognitive demand differences; self versus others) between the children's own choices and the projected mom's choices. However, although one might expect differences in the dorsomedial prefrontal cortex (PFC) or temporo-parietal junction (TPJ) that often associated with reflected self-appraisals or social perception^18^,^19^, we did not observe any statistically significant difference between the two event indicator regressors at our whole-brain threshold (*P*<0.05 corrected; Supplementary Fig. 1).

**Figure 4:**
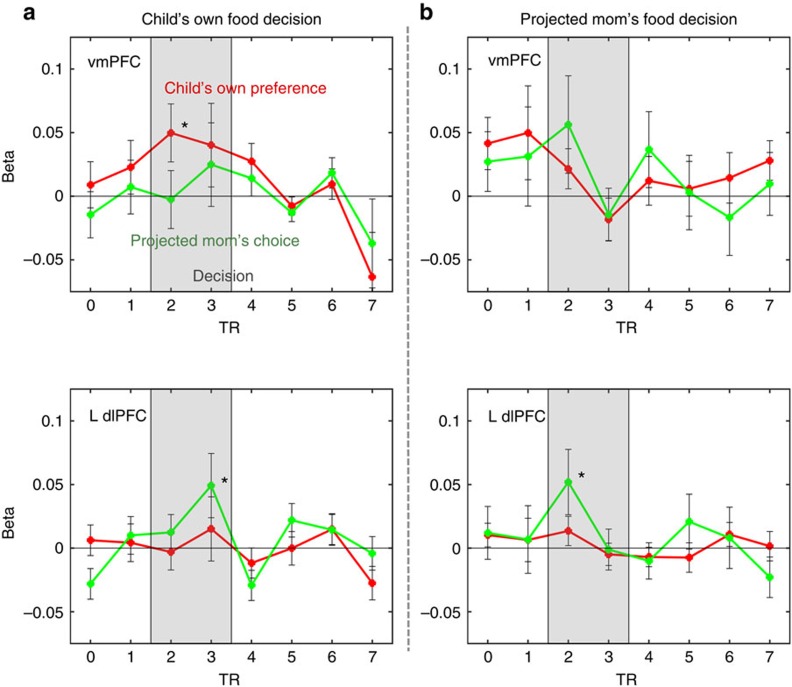
vmPFC and dlPFC ROI time series. (**a**) ROI time series of the beta-weights of parametric regressors for child's own food preferences and the projected mom's choices in my choice trials. (**b**) ROI time series of the beta-weights of parametric regressors for child's own food preferences and the projected mom's choices in mom's choice trials. TR=2.53 s. The grey box represents a visual aid for the approximate decision period, adjusted for the fMRI haemodynamic response lag. All error bars denote s.e. *P<0.05 (one-sample *t*-tests against zero).

Next, we further explored how the neural correlates of two decision-related signals changed over the course of decision time in the vmPFC and left dlPFC regions by constructing the time-series beta-weight (effect size) plots of the parametric regressors (Fig. 4). As described in the methods, we conducted GLMs with a finite impulse response (FIR) basis functions for 7 repetition time (TR)s (∼18 s) from the onset of stimuli. At the time of children's own choices, the vmPFC activity that correlated with own preferences showed a significant effect (*t*_24_=2.20, *P*<0.05) after 2 TRs from the onset of stimuli (∼5 s), whereas the left dlPFC activity that correlated with the projected mom's choices showed a significant effect (*t*_24_=2.28, *P*<0.05) after 3 TRs (∼7.5 s). Interestingly, at the time of the projected mom's choices, the left dlPFC activity revealed a significant effect (*t*_24_=2.10, *P*<0.05) after 2 TRs (∼5 s). None of other time points showed a statistically significant effect. Even though there is an inherent limitation of the temporal resolution of fMRI blood oxygenation level-dependent (BOLD) data, the delayed effect of the dlPFC signal relative to the vmPFC signal we observed at the time of children's own choices suggests a potential temporal difference of integrating two decision-related signal inputs into children's food decisions.

We also ran the GLM-2 to confirm that the left dlPFC activity observed in the previous GLM-1 represent the projected mom's choices, not health values. In the GLM-2, the parametric regressor of children's health ratings from the behavioural task was entered instead of the projected mom's choices along with other regressors of non-interest. As before, brain activity in the vmPFC positively correlated with children's own preferences at the time of children's own choices (*P*<0.05 corrected), but not at the time of the projected mom's choices (Table 2). Most importantly, the left dlPFC region did not show a significant correlation with health ratings at our predetermined whole-brain threshold (*P*<0.05 corrected), suggesting the dlPFC signals were better represented by the projected mom's choices rather than the health attributes. The robustness of our findings that children brain encodes the projected mom's choice at the time of own choices was further supported by supplementary analyses that included GLM-S1 (Supplementary Note 1) with only taste and healthy ratings as predictors (Supplementary Table 2) and GLM-S2 (Supplementary Note 2) with all four ratings as predictors (Supplementary Table 3).

**Figure 5:**
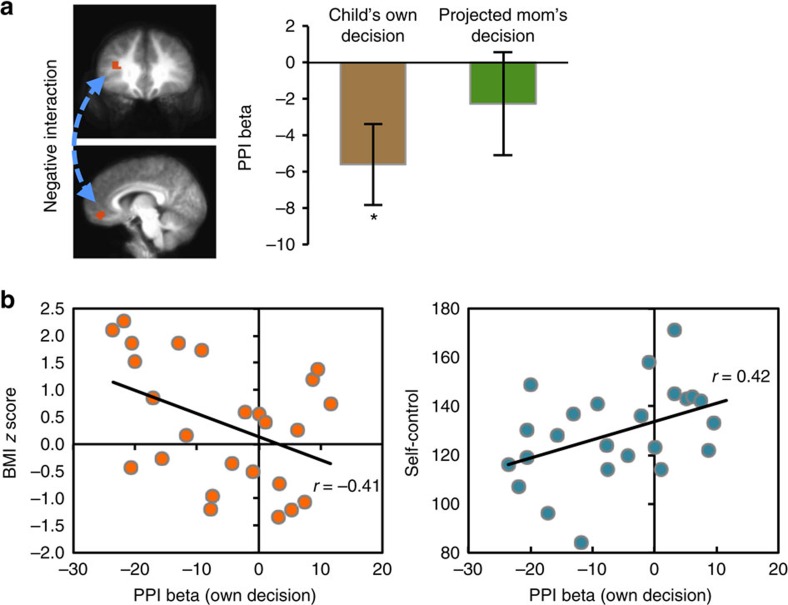
Functional connectivity analysis. (**a**) vmPFC area showed significant negative functional connectivity with left dlPFC in own choice trials, but not in mom's choice trials. ROIs are shown in red. All error bars denote s.e. *P<0.05. (**b**) The beta-weights of functional connectivity between the left dlPFC and vmPFC in own choice condition negatively correlated with the BMI *z*-scores and positively correlated with the self-control scores.

Finally, we investigated the task-related functional connectivity between the vmPFC and left dlPFC brain areas by performing a psychophysiology interaction (PPI) analysis. We used the left dlPFC as a seed region, as it showed significant activations during both choice conditions. The left dlPFC region revealed significant negative functional connectivity with the vmPFC during own choice trials (*t*=−2.50, *P*<0.05, Fig. 5), suggesting an inhibitory relationship between the children's own preference values and the projected mom's decision values at the time of children's own food decisions. Not surprisingly, the left dlPFC and vmPFC regions showed no significant functional connectivity during the projected mom's choice trials, suggesting these two regions significantly interact only when children made their own choices. We further postulated that if the inhibitory functional interaction between the dlPFC and vmPFC modulates children's own food choices, its connectivity strength would be correlated with children's body mass or their ability to exercise self-control. To test this possibility, we performed correlational analyses across subjects. Stronger inhibitory functional connectivity between the dlPFC and vmPFC was significantly associated with higher BMI *z*-scores (*r*=−0.41, *P*<0.05) and lower self-control scale scores (*r*=0.42, *P*<0.05). The correlation between BMI *z*-scores and self-control scores was not significant (*r*=−0.31, *P*=0.14). In our results, children with excessive body mass or low self-control scores showed stronger inhibitory functional connectivity between the left dlPFC and vmPFC regions at the time of their own food choices.

## Discussion

Our behavioural and fMRI results demonstrate novel empirical evidence that children use different computational and neurobiological mechanisms when they make their own food choices, compared with when they estimate their mothers' food choices for them. Not surprisingly, children's own food choices were solely predicted by taste values, revealing their strong preference for delicious foods, and their de-emphasis on health values. When children selected foods they believed their mother would choose for them, they used health values as well as taste values. Therefore, it appears that children in our sample were aware of the health aspects of the foods, but had difficulty incorporating these values into their own choices, or could not assign priority to health over taste. Nonetheless, children could compute and utilize their projected mom's choices while making their own food choices. This suggests asking children to consider what a parent would want them to eat might help them make better food decisions.

In our fMRI data analyses, we found that the children's own preferences are encoded in the vmPFC, whereas the projected mom's choices are encoded in the left dlPFC, just as we hypothesized. The vmPFC is a critical region for goal value representation^15^,^17^,^20^. The dlPFC areas identified in our results are similar to areas that are known to play critical roles in self-control^7^,^8^ and social cognition^21^. Furthermore, the dlPFC region revealed an inhibitory (negative) functional connectivity with the vmPFC at the time of children's own choice in a way tightly linked with related findings in adults^6^,^8^,^22^. It is worth noting that children's brain recruits the same vmPFC–dlPFC circuit previously identified in adults^6^,^8^,^22^ to make their own dietary choices, while children's decisions are regulated by different value attributes. In our study, the left dlPFC did not show any significant correlation with health values in either the children's own choice or the projected mom's choice conditions at our whole-brain threshold, whereas previous adult studies showed the left dlPFC activity correlated with health values^8^. Instead of health values, the left dlPFC encoded the projected mom's food choices. These results suggest that the same left dlPFC region that encodes the projected maternal choices in children may serve a similar modulatory role on food decisions in both children and adults, but use different value information inputs (that is, children using projected maternal choices versus adults using healthiness attributes) during decision-making. Another point to further consider would be a potential difference of modulatory signals originating from the dlPFC region between children and adults' dietary choices. In the self-controller group (adults who used both taste and heath values for their decision) of Hare *et al*.^6^, the health values could be considered as intrinsically motivated signals (that is, these health values positively contributed for self-controller's own voluntary decision). However, the modulatory signals in the children's dietary choices in the form of the projected mom's choices might not be intrinsically motivated signals by themselves (beside of psychological motivational effects in child–mother relationships). Actually, in our behavioural choice data, health values were negatively correlated with children's own preferences (*r*=−0.17, *P*<0.05), whereas they were positively correlated with the projected mom's choices (*r*=0.55, *P*<0.01). Thus, we can speculate that children utilized the internalized mother's choices to modulate their own choices, even when they personally do not like some healthy food items. Even with this potential difference in the nature of modulatory signals, our study demonstrated that the vmPFC–dlPFC circuits cooperates in a similar modulatory manner in both children's and adults' dietary choices. Also, in our study the same left dlPFC region encoded the projected mom's choices in mom's trials in which children's self-regulation was not required. In addition, the left dlPFC did not show a significant functional connectivity with the vmPFC. Taken together, we speculated that the contribution of the left dlPFC to the self-regulated decision process is context-dependent rather than omnipresent.

Interestingly, the time-series beta-weight plot of parametric regressors revealed temporally separated peaks of the vmPFC and left dlPFC activities during children's own food decision-making process. Although fMRI data cannot provide precise temporal information beyond the 1 TR unit (2.53 s) difference between two peak correlations, our findings are consistent with a recent behavioural report^23^ that argued that tastiness attribute information (primary reward signals) is incorporated into the food decision process earlier than healthfulness attribute information (regulatory signals) in adults. Sullivan *et al*.^23^ also proposed that the longer delay between taste and health attribute signals is critically related to self-control failures of food choices^23^. As stated earlier, health values did not predict children's food decisions. Instead, a regulatory decision-related signal as a form of the projected maternal choices may contribute to children's food decision process in a later stage after an early preference-based taste value encoding. Furthermore, the left dlPFC region showed a peak correlation after 2 TRs from the onset of stimuli in mom's choice trials, which was 1 TR unit faster than children's own choice trials. Thus, our data further support the two-stage process of food decision-making in which the effortful regulatory signals emerge relatively later (when they are needed), after the initial anticipated reward or preference-based signals. However, future research with more precise temporal resolution will be required to elucidate the exact timing of children's food decisions and its implication on self-regulation development in children.

Prior research on reflected appraisals (that is, person's self-perception of how others evaluate or see him or her) or social perception^18^,^19^ shows that the dorsomedial PFC, TPJ or posterior cingulate regions are recruited when participants make judgments about ‘self' and ‘others.' However, we did not observe any statistically significant effect in the comparison between two choice event conditions. We think our experimental task is fundamentally different from those studies, which makes it hard to directly compare our study with them. In this study, children participants were asked to make a series of ‘value-based decisions' (eat or not eat) about food items (external objects that are not directly linked to self-perception), and they were not asked to make inferences about self's or other's internal states, intention or beliefs. Thus, we did not expect neural activities in social perception or reflected self-appraisal-related regions such as medial PFC, TPJ or posterior cingulate. Indeed, our findings are highly consistent with the recent decision neuroscience studies^6^,^7^,^8^ that demonstrate that vmPFC and dlPFC play central roles for self-regulation in value-based decision-making.

We believe that the computation and utilization of the projected caregiver's choices at the time of the child's own choices represent key developmental aspects of a child's value-based decisions. These are fundamentally distinct from those of adults. The projected mom's choices may play a critical modulatory role in children's choices more generally. In early child–caregiver relations, children are motivated to monitor and regulate their behaviours to satisfy their caregivers' expectations^24^. Thus, parental influences must play a crucial regulatory role in a variety of children's health decision-making scenarios during the maturation of self-regulation functions. However, as children enter into adolescence and young adulthood, their decisions become more independent from their parents', and peers may be more influential^25^. Interestingly, recent studies show that adolescent risk-taking behaviours increase in the presence of peers^26^ and are reduced in the presence of mothers^27^. Thus, it will be worth investigating how the computational and neurobiological decision mechanisms demonstrated in this study change as children develop through adolescence and early adulthood.

Our first-of-a-kind study successfully demonstrated that children compute and utilize the projected caregiver's choices at the time of their own choice. However, there are several caveats that this study could not fully explain. First, as mentioned above, it would be important to better understand a developmental trajectory of children's decision-making mechanisms. In our fMRI results, participants between the ages of 8 and 14 years, age or pubertal development did not show a statistically significant correlation with behavioural food choices. However, given a relative small sample size of this fMRI study, it would be beneficial to systematically investigate a full developmental trajectory of children's decision-making mechanism with larger samples that include a broader age range. In particular, it would be informative to examine when the projected maternal choices first begin to influence children's own decision-making in younger children. A recent study showed that preschoolers (3–5.5 years old) rated food items less tasty and were less likely to eat, when food was presented as instrumental to achieve health goals^28^. Thus, it is unsure whether younger children under age of 8 utilize a similar decision mechanism to that found in this study. Second, another crucial question to be considered is how meaningfully our findings could be generalized to children's real-life food decisions (for example, children's food choices at school cafeteria or home), different sources of social influences (for example, influences of father, siblings, peers or teachers; traditional or non-traditional parental roles; and cultural backgrounds) or different non-food types of children's value-based choices (for example, playing video games or reading books; and whom to invite to a birthday party) beyond the experimental conditions we manipulated. Future studies that expand ecological validity and generalizability are necessary for real-life interventions, as well as scientific verifications.

Raising children to make healthy and effective decisions is of paramount importance. Our results provide important insights into how to promote healthy food choices among children. In turn, promotion of healthy food choices may prevent excess weight gain and its concomitant medical and psychosocial consequences. Traditional health education approaches designed for adults to incorporate health values for their food decisions may not be effective for children. For instance, basic nutritional information may not be useful for children who cannot fully understand what calories, fat percentage, cholesterol and processed sugars mean^29^. Furthermore, children are heavily exposed to marketing advertisements that promote unhealthy food consumption^30^, which make children's healthy food choices even more difficult^31^. Our findings emphasize the pivotal role of caregivers in promoting children's food choices. The foundational food preferences shaped in early years likely persist. How parents choose foods for their children in early childhood can shape the eating behaviours of their children as they age^32^,^33^. Parents should be primary targets of intervention programmes to promote healthy eating behaviours among children, reducing the risk of paediatric obesity.

In general, the computational and neural mechanisms of children's decision-making are relatively poorly understood. Children may compute and use their projected caregiver's preferences as an internalized behavioural regulator in a variety of food and non-food choice situations. Also, previous literature suggests that self-regulation can be transmitted across generations through child–parent relationships^34^. Much scientific work is required to better understand the neurobiological and behavioural mechanisms of paediatric decision-making, particularly in the context of child–parent relations. An improved understanding of how children make decisions is imperative for developing age-appropriate interventions that promote a lifetime of healthy choices.

## Methods

### Participants

Twenty-five children between the ages of 8 and 14 (mean=10.9 years old; 14 boys; 18 Caucasian, 1 African American, 2 Hispanic, 1 Asian and 3 Multiracial) completed our experiment. We chose an 8–14-year age range, similar to that used in the study by Bruce *et al*.^35^, to capture middle childhood with most participants before adolescence. During this developmental period, youth are making increasingly independent decisions and it is therefore important to learn more about the process of these decisions. Five additional children and their mothers participated in a subset of the tasks, but were excluded from further analyses due to behavioural or technical problems (excessive head motion > 3 voxels, non-compliance to task instructions or no button response recordings). All children provided assent and their mothers provided written informed consent before participation, as approved by the Human Subjects Committee of the University of Kansas Medical Center. We choose to recruit only mothers for experimental control purposes, considering that mothers typically play a primary role on early child-feeding practices^36^. Participants were in good health, right-handed, had normal or corrected-to-normal vision, had no history of attention deficit hyperactivity disorder, other psychiatric diagnoses, or neurological or metabolic illnesses. Children were not taking any psychotropic medications and had no history of allergies to the food items used in the experiment. Children's heights and weights were measured using a stadiometer (Perspective Enterprise, PE-WM-60-84) and scale (Befour, PS6600 ST) to calculate BMI (kg m^−2^). The BMI scores were converted to age- and gender-adjusted BMI *z*-scores. Children also completed the self-administered rating scale of pubertal development^37^ and self-control scale^38^.

### Procedures

Sixty food images were used for the behavioural food-rating task and fMRI food-decision task. Children completed all tasks in the absence of their mothers. Stimuli included items often consumed by children, and were selected to include a wide range of tastiness and healthiness attributes (for example, apple, broccoli, asparagus, glazed donut, French fries and marshmallows). All food pictures were high-resolution (72 d.p.i.) colour images with a size of 300 × 300 pixels. The stimulus presentation and behavioural response collection were controlled by Presentation software (Neurobehavioral System).

### Behavioural food-rating task

Before beginning the food-rating task, the food images were introduced by the research staff to ensure identification of any unfamiliar foods. To confirm participants understood the food-rating tasks, the task type was cued by an initial instruction display (taste-rating task or health-rating task) and a four-point rating scale (very bad–very good or very unhealthy–very healthy) was presented below the food image during the rating decision process. Child participants first provided separate ratings for taste attributes (very bad, bad, good or very good) and health attributes (very unhealthy, unhealthy, healthy or very healthy) for each food item presented on a laptop monitor using buttons on the keyboard. Participants provided ratings in two separate tasks (a taste-rating task and a health-rating task) and the order of the two tasks was counterbalanced across participants. For each food item, children were instructed to provide their own taste rating regardless of health attributes and provide their own health rating regardless of taste attributes. Participants were then asked to provide a liking rating for each food item indicating how much they liked or disliked the item using a five-point rating scale (strongly dislike, dislike, neutral, like and strongly like). This preference-rating task was always presented after the taste and health ratings. For all rating runs, food items were presented in a random order and stayed on the screen until a response was given. Rating trials were separated by a 1-s fixation cross screen.

### fMRI food-decision task

Inside the MRI scanner, child participants engaged in the food-decision task (total scanner time of ∼70 min). The food-decision task included two different types of decision condition blocks (‘my choice' condition and ‘mom's choice' condition) that were randomly presented. In the my choice condition, children were asked to make decisions about how much they want to eat the food item presented on the screen. Children were instructed and encouraged to make these decisions as real choices, because one of their own choices (not from mom's choices) would be randomly selected, and the food item chosen would be given to them to eat at the end of the fMRI experiment. In the ‘mom's choice' condition, children were asked to guess their mom's food choices for them (that is, would she want you to eat this food?) using the same scale. Child participants were instructed that they should enter the projected mom's choice for them, not what the mom would choose for herself. Participants completed four runs of the decision task and each run included three my choice blocks and three mom's choice blocks. Each choice block included 10 decision trials. The order block and the order of trials within the block were randomized. Participants completed a total of 120 decision trials in the ‘my choice' condition and 120 decision trials in the ‘mom's choice' condition. To help children's understanding, the block type was cued by an initial 2-s instruction display (‘my choice' or ‘mom's choice') and the block type indicator was presented above the food image during a decision period. Participants were asked to enter their decisions using a four-point scale (strong no, no, yes or strong yes) during a maximum time limit of 4-s. The food image disappeared after the decision response. The decision scale was presented below the food image. To exclude motor-related responses of no interest in fMRI data analyses, the response button mapping (strong no–strong yes or strong yes–strong no) was counterbalanced across participants. Decision trials were separated by a fixation cross screen of random duration (uniform: 1–5 s).

To increase the ecological validity of the task (that is, granting children's free will for their own choices and minimizing bias for the projected mom's choices), children were informed that one of their ‘own choice' items would be selected at random and they would be given this food item on completion of the study.

### MRI data acquisition

Anatomical and functional scans were acquired using a Siemens 3T Magnetom Skyra scanner (Siemens Medical Systems, Germany) with a 12-channel head coil at the Hoglund Brain Imaging Center of University of Kansas Medical Center. Structural images were acquired first with a high-resolution MPRAGE anatomical sequence (1 mm isotropic voxel; 256 mm field of view). Next, BOLD contrast functional images were acquired with gradient-echo echo-planar T2*-weighted imaging. To optimize functional sensitivity of signals in the orbitofrontal cortex, T2* images were acquired in an oblique orientation of 35° to the anterior commissure–posterior commissure line^39^. Each functional volume consisted of forty-eight axial slices (TR=2,530 ms; echo time (TE)=25 ms; flip angle (FA)=90°; field of view (FOV)=192 mm; 64 × 64 matrix; 3 mm isotropic voxel). A total of 640 volumes were acquired through four functional runs.

### fMRI data preprocessing

Analysis of fMRI data was performed using the AFNI package^40^ as well as custom MATLAB scripts. The first four functional volumes of each run were removed to account for the equilibration effects of magnetization. The following processing steps were applied sequentially: slice-time correction; motion correction; spatial resampling (3 × 3 × 3 mm) and normalization to the standard Talairach template; Gaussian spatial smoothing (full-width at half-maximum: 6 mm); and intensity normalization (each voxel's mean was set to 100).

### Statistical analyses of fMRI data

We estimated several general linear models (GLMs) of the BOLD responses. All of the models allowed for first-order autoregression and included six motion parameters, constants, and linear time trends for each run as regressors-of-non-interest. A two-stage mixed-effects analysis was performed in which the regression coefficients for each condition of interest were tested across participants via *t*-tests. Two-tailed tests were used for all statistical analyses.

We performed multiple comparison corrections at the cluster level using Monte Carlo simulations with the AlphaSim programme (http://afni.nimh.nih.gov). Statistical inferences at the whole-brain level were carried out at a corrected threshold of *P*<0.05 by imposing a *P*<0.005 statistical threshold and a minimum cluster extent of 49 voxels. For pre-determined regions of interest, we performed small volume corrections at the cluster level (*P*<0.005 and extent threshold of 14 voxels for vmPFC and 16 voxels for dlPFC). The anatomically defined mask for the vmPFC region consisted of medial orbital gyrus, rectal gyrus and olfactory cortex masks of AFNI's standard anatomical brain. The anatomically defined mask for dlPFC region consisted of superior frontal gyrus and middle frontal gyrus. Activation coordinates are reported using Talairach coordinates^41^.

*General linear model*. GLM-1: We estimated the GLM-1 on all of the choice trials to identify brain regions that encode children's own preferences and the projected mother's choices at the time of children's food decision. The statistical model included the following regressors: (1) an indicator function (1 for events, 0 otherwise) for the children's choice period (with a duration from stimulus onset to the decision); (2) the indicator function for the children's choice period multiplied by the children's own preferences (measured through behavioural rating trials with identical food stimuli); (3) the indicator function for the children's choice period multiplied by the projected mom's choices (measured through mom's choice trials with identical food stimuli); (4) an indicator function (1 for events, 0 otherwise) for the mom's choice period (with a duration from stimulus onset to the decision); (5) the indicator function for the mom's choice period multiplied by the children's own preferences (measured through behavioural rating trials with identical food stimuli); and (6) the indicator function for the mom's choice period multiplied by the projected mom's choices (measured through the children's responses). The GLM-1 also included missed trials as a regressor of non-interest. Both indicator and parametric functions were convolved with a canonical haemodynamic response function (HRF). The contrasts of interest in this GLM were the four parametric regressors, which can be used to identify brain regions that encode the decision variables we hypothesized.

GLM-2: We estimated the GLM-2 on all of the choice trials to identify brain regions that encode children's own preferences and health attribute values at the time of children's food decision. The GLM-2 included the following regressors: (1) an indicator function (1 for events, 0 otherwise) for the children's choice period (with a duration from stimulus onset to the decision); (2) the indicator function for the children's choice period multiplied by the children's own preferences; (3) the indicator function for the children's choice period multiplied by the health ratings (measured through behavioural rating trials with identical food stimuli); (4) an indicator function (1 for events, 0 otherwise) for the mom's choice period (with a duration from stimulus onset to the decision); (5) the indicator function for the mom's choice period multiplied by the children's own preferences; and (6) the indicator function for the mom's choice period multiplied by the health ratings. The GLM-2 included missed trials as a regressor of non-interest. Both indicator and parametric functions were convolved with a canonical HRF.

*Regions of interest time-series beta-weight analysis*. To further explore how beta-weight (effect size) of parametric regressors of interest varied over time, we constructed time-series graphs for the beta-weights within the vmPFC and left dlPFC regions of interest (ROIs) for the parametric modulators of GLM-1. We defined independent functional ROIs by using a leave-one-subject-out approach^42^. In particular, to define the ROI used to compute the ROI statistics for subject *i*, we estimated the GLM of interest using only the data from all other subjects except the subject *i*. For the ROI definition contrast, the main effect contrast (both my choice trials and mom's choice trials) of the previous GLM-1 (group-level peak coordinates, left dlPFC: *x*=−31, *y*=29, *z*=17; vmPFC: *x*=8, *y*=44, *z*=−7) was used. We then define ROIs by drawing a 6-mm radius sphere centred at the peak of the group-level contrast for the group (*N*−1=24 subjects) that excludes the subject *i* (independent from the subject *i*). This was repeated for every subject. By using the average time series of the BOLD data within ROIs, we ran additional GLMs with a FIR basis function. To cover a full HRF response model, each trial was modelled for 7 TRs from the onset of the choice screen.

*Functional connectivity analysis*. We estimated a PPI model to explore the relationship between the left dlPFC and vmPFC regions. The goal was to test our hypothesis that these two regions would show stronger connectivity when children make their own choices. We chose the left dlPFC ROI (the same ROI used in the previous FIR analyses), which showed significant activations in both types of choice conditions, as a seed region. The PPI analysis was done in three steps. First, we extracted a spatially averaged time series of BOLD activity for the seed ROI. Nuisance variances associated with drifts of the BOLD signals (constant and linear terms for each run) were removed from the extracted time series, which were then deconvolved using a model of a canonical HRF^43^. Second, for each individual, we estimated a GLM-3 with the following regressors: (1) the extracted time series from the ROI (seed); (2) an indicator function of the children's own choice trials; (3) an indicator function of the children's projected mom's choice trials; (4) an interaction (PPI_my choice_) between the deconvolved ROI (seed) signal and an indicator function of the children's own choice trials; and (5) an interaction (PPI_mom's choice_) between the deconvolved ROI (seed) signal and an indicator function of the projected mom's choice trials. The GLM-3 also included all the parametric regressors of the GLM-1. Third, we performed second-level analyses by carrying out *t*-tests on the first-level average contrasts of the PPI regressors within the vmPFC ROI at *P*<0.05 (two-sided). Note that this contrast examines functional connectivity between the left dlPFC and vmPFC ROIs during decision-making.

### Data availability

Owing to data privacy regulations imposed by the Social Sciences IRB of the University of Missouri—Kansas City (UMKC) and the Human Subjects Committee of the University of Kansas Medical Center (KUMC), data cannot be made publicly available. Data are available on request from the corresponding author.

## Additional information

**How to cite this article:** Lim, S.-L. *et al*. The child brain computes and utilizes internalized maternal choices. *Nat. Commun.* 7:11700 doi: 10.1038/ncomms11700 (2016).

## Supplementary Material

Supplementary Information Supplementary Figure 1, Supplementary Tables 1 - 3 and Supplementary Notes 1 and 2

## Figures and Tables

**Table 1:** Brain regions correlated with children's own preferences and projected mom's choices in the food decision task (GLM-1).

**Region**	**L/R**	**Talairach**			***t***
		***x***	***y***	***z***	
*Own choice condition*					
Children's preferences					
vmPFC	L/R	2	41	−7	3.90^svc^
Projected mom's choices					
dlPFC	L	−31	29	17	4.30
Precentral gyrus/inferior frontal gyrus	L	−34	−1	29	4.30
					
*Mom's choice condition*					
Children's preferences					
Middle occipital gyrus	L	−37	−76	−1	−4.13
	R	29	−85	−16	−4.87
Projected mom's choices					
dlPFC	L	−43	32	23	3.47^svc^
Middle temporal gyrus	L	−55	−55	−1	4.87

dlPFC, dorsolateral prefrontal cortex; vmPFC, ventromedial prefrontal cortex.

*P*<0.05 with whole-brain cluster size correction (height threshold *t*=3.09, *P*<0.005; extent threshold *k*=49 voxels); ^svc^, *P*<0.05 with small volume correction (height threshold *t*=3.09, *P*<0.005; extent threshold *k*=14 voxels for vmPFC and 16 voxels for dlPFC).

**Table 2:** Brain regions correlated with children's own preferences and health ratings (GLM-2).

**Region**	**L/R**	**Talairach**			***t***
		***x***	***y***	***z***	
*Own choice condition*					
Children's preferences					
vmPFC	L/R	5	35	−7	4.10
Health ratings					
None					
					
*Mom's choice condition*					
Children's preferences					
Middle occipital gyrus	L	−5	−83	3	−4.63
Health ratings					
None					

*P*<0.05 with whole-brain cluster size correction (height threshold *t*=3.09, *P*<0.005; extent threshold *k*=49 voxels).
