# Corrigendum: The child brain computes and utilizes internalized maternal choices

**DOI:** 10.1038/ncomms12127

**Published:** 2016-06-23

**Authors:** Seung-Lark Lim, J. Bradley C Cherry, Ann M. Davis, S. N. Balakrishnan, Oh-Ryeong Ha, Jared M. Bruce, Amanda S. Bruce

Nature Communications 7: Article number: 1170010.1038/ncomms11700 (2016); Published: 05242016; Updated: 06232016.

The financial support for this Article was not fully acknowledged. The Acknowledgements should have also included the following:

The Hoglund Brain Imaging Center is supported by a generous gift from Forrest and Sally Hoglund and funding from the National Institutes of Health (P30 HD002528, S10 RR29577, UL1 TR000001). The content is solely the responsibility of the authors and does not necessarily represent the official views of the funding agencies.

