# Supplementary material for: The child brain computes and utilizes internalized maternal choices
Source: Nat Commun. 2016 May 24;7:11700. doi: 10.1038/ncomms11700 (PMC4890300; doi:10.1038/ncomms11700)
Supplement: Supplementary Information — Supplementary Figure 1, Supplementary Tables 1 - 3 and Supplementary Notes 1 and 2 [file ncomms11700-s1.pdf]

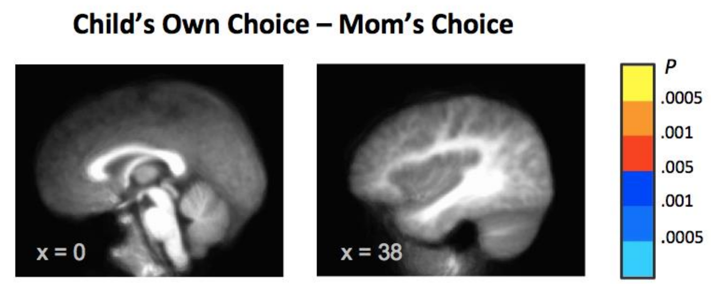

Supplementary Figure 1. Own choices versus mom's choices. Two event indicator regressors were compared to explore potential systematic task differences (e.g., motivational or cognitive demand differences; self vs. others) between children's own choices and the projected mom's choices. No statistically significant difference was observed at our whole-brain threshold (*P* < .05 corrected).

Supplementary Table 1. Average zero-order Pearson's correlation coefficients among behavioral ratings and descriptive statistics (*N* = 25)

**Behavioral Ratings**	Children's own choice	Projected mom's choice	Preference ratings	Taste ratings	Health ratings
1. Children's own choice	1				
2. Projected mom's choice	0.20* (.46)	1			
3. Preference ratings	0.65** (.24)	0.10 (.34)	1		
4. Taste ratings	0.70** (.18)	0.06 (.39)	0.68** (.25)	1	
5. Health ratings	-0.13 (.40)	0.55** (.35)	-0.17* (.34)	-0.22** (.35)	1
Rating *M (SD)*	2.88 (.25)	2.75 (.26)	3.69 (.37)	2.67 (.21)	3.02 (.20)

* *P* < .05; ** *P* < .01. Average correlation coefficients and standard deviations. For each individual, zero-order correlations were separately computed first. Then, group-level analyses were performed through one-sample *t*-tests against zero. 1 and 2 ratings are children's decision responses (4-point scale) acquired during fMRI scans. 3, 4, and 5 ratings (5-point scale) were acquired before fMRI scans.

Supplementary Table 2. Brain regions correlated with the taste and health ratings in food decision task (GLM-S1).

Region	L/R	Talairach			*t*
		x	y	z	
**Own choice condition**					
**Taste ratings**					
Ventromedial Prefrontal Cortex (vmPFC)	L/R	5	35	-7	4.88
**Health ratings**					
None					
**Mom's choice condition**					
**Taste ratings**					
None					
**Health ratings**					
None					

$P < .05$ with whole brain cluster size correction (height threshold $t = 3.09$, $P < .005$; extent threshold $k = 49$ voxels).

Supplementary Table 3. Brain regions correlated with children's own preferences, the projected mom's choices, taste ratings, and health ratings in the food decision task (GLM-S2).

Region	L/R	Talairach			*t*
		x	y	z	
**Own choice condition**					
**Children's preferences**					
Ventromedial Prefrontal Cortex (vmPFC)	L/R	2	41	-7	3.96[svc]
**Projected mom's choices**					
Dorsolateral Prefrontal Cortex (dlPFC)	L	-31	29	17	4.10
Precentral Gyrus / Inferior Frontal Gyrus	L	-34	2	29	4.26
**Taste ratings**					
None					
**Health ratings**					
None					
**Mom's choice condition**					
**Children's preferences**					
Middle Occipital Gyrus	L	-7	-85	-1	-4.75
	R	11	-85	-1	-4.53
**Projected mom's choices**					
Dorsolateral Prefrontal Cortex (dlPFC)	L	-40	32	23	3.43[svc]
Middle Temporal Gyrus	L	-55	-55	-1	4.80
**Taste ratings**					
None					
**Health ratings**					
None					

$P < .05$ with whole brain cluster size correction (height threshold $t = 3.09$, $P < .005$; extent threshold $k = 49$ voxels); [svc], $P < .05$ with small volume correction (height threshold $t = 3.09$, $P < .005$; extent threshold $k = 14$ voxels for vmPFC and $k = 16$ voxels for dlPFC).

Supplementary Note 1

General linear model (GLM)-S1: On all of the choice trials, we estimated the GLM-S1 in which children's own preferences and health attribute values were simultaneously entered into the model. The statistical model included the following regressors: (1) an indicator function (1 for events, 0 otherwise) for the children's choice period (with a duration from stimulus onset to the decision), (2) the indicator function for the children's choice period multiplied by the children's own preferences (measured through behavioral rating trials with identical food stimuli), (3) the indicator function for the children's choice period multiplied by the health ratings (measured through behavioral rating trials with identical food stimuli), (4) an indicator function (1 for events, 0 otherwise) for the mom's choice period (with a duration from stimulus onset to the decision), (5) the indicator function for the mom's choice period multiplied by the children's own preferences (measured through behavioral rating trials with identical food stimuli), and (6) the indicator function for the mom's choice period multiplied by the health ratings. The GLM-S1 also included missed trials as a regressor of non-interest.

Supplementary Note 2

General linear model (GLM)-S2: We estimated the GLM-S2 in which children's own preferences, projected mom's choices, taste ratings, and health ratings were entered simultaneously. The GLM-S2 included the following regressors: (1) an indicator function (1 for events, 0 otherwise) for the children's choice period (with a duration from stimulus onset to the decision), (2) the indicator function for the children's choice period multiplied by the children's own preferences, (3) the indicator function for the children's choice period multiplied by the projected mom's choices, (4) the indicator function for the children's choice period multiplied by the taste ratings, (5) the indicator function for the children's choice period multiplied by the health ratings, (6) an indicator function (1 for events, 0 otherwise) for the mom's choice period (with a duration from stimulus onset to the decision), (7) the indicator function for the mom's choice period multiplied by the children's own preferences, (8) the indicator function for the mom's choice period multiplied by the projected mom's choices, (9) the indicator function for the mom's choice period multiplied by the taste ratings, and (10) the indicator function for the mom's choice period multiplied by the health ratings. The GLM-S2 included missed trials as a regressor of non-interest. Similar to the behavioral regression model, the taste and health attribute value regressors were orthgonalized in respect to both children' own choices and projected mom's choices regressors to avoid a potential multicolinearity issue.
